# 

**Published:** 1856-06

**Authors:** 


### CONTENTS.

***ORIGINAL COMMUNICATIONS.***

PAGE

ARTICLE I.—Small Pox as it appeared in Richmond, Va., and its vicinity in the winter of 1855 and 1856. By THOMAS POLLARD, M. D., of Richmond, Va. ........ 577

ARTICLE II.—A Case of Partial Placenta Prævia, complicated with transverse presentation and rigidity of the Os Uteri. By M. J. GREENE, M. D., of Loachapoka, Ala. ........ 591

***SELECTIONS.***

Medical Jurisprudence.—Insanity in relation to Crimes ........ 601

On the Practical Application of Chloroform as a Topical Anæsthetic to Mucous and Cutaneous Surfaces ........ 610

Memoir on the Simple Ulcer of the Stomach ........ 615

An Unique Case of Dislocation of the Patella.—Complete Revolution of the Bone on its Long Axis ........ 620

Simple and Puerperal Peritonitis ........ 624

***EDITORIAL AND MISCELLANEOUS.***

Atlanta Medical College ........ 630

To Contributors ........ 631

Bibliographical ........ 632

Abstract of the Proceedings of the Ninth Annual Session of the American Medical Association, held in Detroit, May 6, 7, 8 and 9, 1856 ........ 636

